# Account of a Patient Who Swallowed a Set of Artificial Teeth

**Published:** 1858-07

**Authors:** Paul F. Eve


### ARTICLE XVII.

*Account of a Patient who Swallowed a set of Artificial Teeth.* A paper read before the Tennessee State Medical Society, April 7, 1858. By Paul F. Eve, M. D.

At 3 A. M., 30th March, was called up to Mr. B., aged 23, who came to my office to have removed from his œsophagus, if possible, a set of three teeth on a gold plate, which he had swallowed a few minutes previously while asleep. They are the two central and the right lateral incisors, fixed on a broad plate of gold, having on air chamber to secure the piece on the atmospheric principle, to the roof of the mouth. They measure one inch and a half in one diameter, and one inch and three-tenths in another.

The patient indicated the usual site of arrest for foreign substances lodged in the gullet, viz. the union of the pharynx with the œsophagus. Attempts were first made to ascertain its position by an ivory ball at the end of a whalebone rod, and then to remove it by forceps and the hook or silver basket of Dupuytren. No metallic ring or click was produced by these instruments, though the plate or teeth were seized once or twice. While all violent efforts were avoided, they were nevertheless continued for several minutes. There was a thick viscid secretion hawed up, and it was colored with blood once or twice after removing the forceps, but there was no hemorrhage, nor did the patient complain or object to any of the proceedings. An emetic of ipecacuanha, with copious draughts of warm water, were now administered, and in the vomiting produced, the foreign body was fortunately ejected.

March 30th, 8 A. M., found the patient cheerful, but of course, having great difficulty of deglutition, some tumefaction of the throat, and soreness down the œsophagus. He had slept a little. Prescribed ice, and iced-gum tea exclusively, and to keep in bed. At 3 P. M., gave an ounce of sulphate of magnesia in mucilage, in divided doses. It commenced to operate at 10 o'clock, and acted freely on his bowels.

31st. Is better. Allowed thin arrow root with orangeade.

April 1st. Is doing well, and permitted to leave his room, situated over the store, in a close carriage, for his employer's residence. Had little or no fever. Takes the arrow root boiled in milk.

April 2d. Has been sitting up by the fire occasionally today, and seems out of danger. Uses still a limited diet, and that only of the blandest nature. His bowels are in a good condition.

April 3d. Called to patient at 5 A. M. Has apparently taken a violent cold, and to confirm this opinion his friends tell me they found him sitting up last evening by a fire, but without shoes or socks. He had had the day before some bleeding from the nose, to attacks of which he was liable, and to arrest which he applied cold water freely to his face and neck, and drew it up his nostrils. He occupied a small room having two doors on opposite sides to the fire place, with a window adjoining it. The weather too was quite cold on the 1st and 2d of April. He had now considerable difficulty of breathing, talked as one laboring under catarrh, and said he could not swallow. These symptoms had been increasing in violence for three or four hours. Gave immediately ipecacuanha, and even repeated the ordinary dose of it twice afterwards, but without exciting vomiting. This medicine purged him freely during the day, and the next night he had two or three small actions. By examining his throat with a good light while the patient was seated before an open window, the uvula was found much elongated and œdematous, the tonsils enlarged, and the whole pharynx inflamed. This was made at five o'clock in the afternoon of this day. Pulverized alum, cayenne pepper in gargle, and ice were used internally, and a blister applied to the anterior region of the neck externally. His fever, which was high, abated towards evening, he spoke and swallowed easier, and said he felt better. Six visits were made this day.

April 4th, 12½ A. M. Called to patient. Found him worse. The respiration is very laborious, with much mucus in the throat, but which he still could spit out well. He complained of acute pain in his chest, particularly on the left side. Applied mustard plasters on both sides, and then down the thoracic spinal column, which last blistered. Left him at two o'clock, with instructions if he got no better to send for me again, and at the same time to call for Dr. Bowling or Dr. Jennings. Heard nothing further until my visit at 8 A. M., when observing no improvment, and the pupil of his eyes dilating, I called for counsel, and promised to return as soon as I could see one or two patients, and to stay by him during the day, so as to be ready to open the wind-pipe should he suffocate. Returned ten minutes to 9 o'clock, when the patient was in *articulo mortis.* The larynx was instantly laid open with a penknife, and a messenger dispatched for laryngeal tubes. The patient was actually revived, the pupils contracted, but with the returning circulation the blood poured into the wind-pipe. Dr. Bowling came in at this moment; a canula was now introduced, and artificial respiration kept up for about twenty minutes; but the brain was apparently too far paralyzed to carry on the functions of life.

There was not one drop of suppuration about the fauces or mouth, and no evidence whatever of mortification. The patient had lived five full days after the removal of the false teeth and plate. He had recovered pretty well from the injury they had produced, and the efforts made for extracting them, could swallow with ease, respired naturally, and conversed freely on the third and fourth days; only after exposure to cold he had symptoms of œdematous inflamed, or as it is more commonly called, sore throat, and this alone must have destroyed life in his case.

It is proper to remark that Mr. B. was a young man of most exemplary character, had an excellent constitution, and few patients ever co-operated more willingly than he did with every thing proposed for his relief. It was his unfailing cheerfulness that betrayed me into the error of leaving him for a single moment on the morning he died.—*Nashville Jour. of Med. and Sur.*

